# Pioneering *Exploration* for a lasting and sustainable future

**DOI:** 10.1002/EXP.20240397

**Published:** 2024-12-10

**Authors:** Gaopeng Li, Zhi‐Gang Chen, Chengliang Wang

**Affiliations:** ^1^ School of Integrated Circuits Wuhan National Laboratory for Optoelectronics (WNLO) Key Laboratory of Material Chemistry for Energy Conversion and Storage Huazhong University of Science and Technology Wuhan China; ^2^ School of Chemistry and Physics ARC Research Hub in Zero‐emission Power Generation for Carbon Neutrality Centre for Materials Science Queensland University of Technology Brisbane Queensland Australia

To address the growing global imperative for a sustainable future, the United Nations (UN) adopted the 2030 Agenda for Sustainable Development with 17 sustainable development goals (SDGs) at the 2015 UN Sustainable Development Summit. The agenda encompasses 17 SDGs, which span critical areas such as life, water, energy, climate, hunger, poverty, justice, peace, good health, high‐quality education, decent work and economic growth, science and technology. In 2021, *Exploration*, an international, cross‐disciplinary journal, was launched to contribute to these efforts, aiming to inspire and support actionable steps toward a more sustainable future.

In a broad sense, all publications in *Exploration* can be viewed as contributions by researchers and scientists toward a more sustainable world. These contributions encompass: (1) water/salt separation for clean water^[^ ^1^ ^]^; (2) treatment of obsolete water and degradation of plastics for good sanitation; (3) wearable/implantable electronic devices for biomedical applications and good health^[^ ^2^ ^]^; (4) biotechnology and pharmacology for good health,^[^ ^3^ ^]^ for example, advanced biomedical materials for disease diagnosis or therapy and progresses in drug delivery and tumor therapy; (5) energy conversion technologies as actions to achieve affordable and clean energy and reduce the carbon emission,^[^ ^4^ ^]^ such as CO_2_ reduction technologies to convert CO_2_ into organic fuels through electrocatalysis or photocatalysis, hydrogen production from water through electrocatalysis and utilization of solar energy by solar cells or photocatalysis based on perovskites, inverted perovskites, and organics; (6) advanced energy storage technologies with high energy density and long cycle lifespan^[^ ^5^ ^]^ to better harness clean and renewable energies, mitigate the climate change, and make the energy storage devices more convenient and portable.

We firmly believe that sustainable development is essential for the future of humanity. All scientific and technological advancements that contribute to sustainability fall within the scope of this journal and are, therefore, highly encouraged. Beyond the abovementioned cutting‐edge research, we welcome studies in agriculture, electronics, photonics, energies, manufacturing, material science, physical science, environmental science, life science, information science, space science, artificial intelligence, and other fields dedicated to enhancing the quality of life and minimizing humanity's environmental impact. Additionally, topics such as the policies, regulations, and initiatives addressing hunger, poverty, conflicts, and inequalities, aligned with the 17 SDGs, are within our field. Our vision for *Exploration* is to serve as a platform for high‐quality publications across all kinds of disciplines, inspiring researchers in their lifelong commitment to exploring pathways toward a sustainable future.
